# Behavioral and Electrophysiological Correlates of Performance Monitoring and Development in Children and Adolescents with Attention-Deficit/Hyperactivity Disorder

**DOI:** 10.3390/brainsci10020079

**Published:** 2020-02-02

**Authors:** Yanni Liu, Gregory L. Hanna, Barbara S. Hanna, Haley E. Rough, Paul D. Arnold, William J. Gehring

**Affiliations:** 1 Department of Psychiatry, University of Michigan, Ann Arbor, Michigan, MI 48109, USA; ghanna@med.umich.edu (G.L.H.); hannab@med.umich.edu (B.S.H.); hrough@med.umich.edu (H.E.R.); 2 Mathison Centre for Mental Health Research & Education and Department of Psychiatry, University of Calgary, Calgary, Alberta, AB T2N 4Z6, Canada; paul.arnold@ucalgary.ca; 3 Department of Psychology, University of Michigan, Ann Arbor, Michigan, MI 48109, USA; wgehring@umich.edu

**Keywords:** ADHD, performance monitoring, error processing, event-related potentials

## Abstract

The pathophysiology of attention-deficit/hyperactivity disorder (ADHD) involves deficits in performance monitoring and adaptive adjustments. Yet, the developmental trajectory and underlying neural correlates of performance monitoring deficits in youth with ADHD remain poorly understood. To address the gap, this study recruited 77 children and adolescents with ADHD and 77 age- and gender-matched healthy controls (HC), ages 8–18 years, who performed an arrow flanker task during electroencephalogram recording. Compared to HC, participants with ADHD responded more slowly and showed larger reaction time variability (RTV) and reduced post-error slowing; they also exhibited reduced error-related negativity (ERN) and error positivity effects, and reduced N2 and P3 congruency effects. Age effects were observed across groups: with increasing age, participants responded faster, with less variability, and with increased post-error slowing. They also exhibited increased ERN effects and increased N2 and P3 congruency effects. Increased RTV and reduced P3 amplitude in incongruent trials were associated with increased ADHD Problems Scale scores on the Child Behavior Checklist across groups. The altered behavioral and ERP responses in ADHD are consistent with the pattern associated with younger age across groups. Further research with a longitudinal design may determine specific aspects of developmental alteration and deficits in ADHD during performance monitoring.

## 1. Introduction

Attention-deficit/hyperactivity disorder (ADHD) is a neurodevelopmental disorder with symptoms including sustained attention problems, impulsivity, and hyperactivity, affecting ~5% of children and adolescents [1]. Several studies have shown deficits of performance monitoring and adaptive adjustment in children and adults with ADHD [2,3,4]. While some ADHD symptoms may decline from childhood to adulthood in a subset of children [5], the developmental trajectory of performance monitoring and adaptive adjustment along with their underlying neural correlates of the deficits in youth with ADHD remain poorly understood. 

Previous research has shown that children with ADHD perform poorly in a wide range of tasks involving conflict monitoring and inhibitory control (e.g., Go/No-go, flanker, and stop-signal tasks [6]). In general, their behavioral responses in these high cognitive demand tasks tended to be slower, more variable, and more error-prone, and they showed deficits in adaptation to task demands and following error responses [7,8,9]. Post-error slowing, or an increase in RT on trials following an error, is a common behavioral indicator of adaptive control [10]. A failure to slow responding on post-error trials has been interpreted as reflecting a deficit in adaptive control. Diminished post-error slowing was found in children with ADHD, relative to controls [11,12]; however, other studies [13,14] reported intact post-error slowing in children with ADHD compared to typically developing children using a flanker task. 

In studies using event-related potential (ERP) measures, children with ADHD have also shown deficits in neurocognitive processes of response inhibition and performance monitoring. Error-related negativity (ERN) and error positivity (Pe) are two reliable ERP indices of performance monitoring. Both components are time-locked to responses. The ERN has been observed in a variety of tasks; its onset coincides with response initiation, and it peaks 50–100 milliseconds (msec) thereafter [15]. The ERN has a fronto-central distribution, and is believed to be generated in the anterior cingulate cortex and nearby medial frontal regions involved in self-regulation and performance monitoring [15]. The ERN increases with age after early childhood and reflects the activity of a system that detects errors, increases cognitive control, and adjusts behaviors [15]. Following the ERN, the Pe occurs about 200–400 msec after an error and has a centro-parietal distribution, reflecting error awareness, motivational significance of errors, and initiation of adaptive control processes [16]. Some ADHD studies reported reduced ERN in children and adults with ADHD relative to healthy controls (HC) [17,18]; others reported null findings [19,20], and still another reported increased ERN in ADHD [21] (see review papers [6,22]). Pe results in ADHD studies are also mixed: some studies reported diminished Pe [11,13,23,24] (see review [6]), suggesting deficient error valuation or conscious error processing in ADHD, but others reported no difference [21,25] between participants with ADHD and HC. 

In addition to the response-locked ERN and Pe, the N2 and P3 are two main ERP components elicited during stimulus processing, reflecting processes involved in stimulus evaluation and response selection. Specifically, the N2 is a fronto-central negative voltage deflection peaking between 200 and 400 msec after stimulus onset; in cognitive control tasks, a larger N2 is elicited by high conflict trials (e.g., No-go trials in Go/No-go tasks, incongruent trials in flanker tasks) relative to low cognitive conflict trials (e.g., Go trials in Go/No-go tasks and congruent trials in flanker tasks), reflecting adaptive conflict monitoring [26,27,28]. The P3 is a central-parietal positive voltage deflection peaking between 300 and 500 msec after stimulus onset, first observed in an auditory oddball task, which reflects processes related to attention and working memory [29]; it is also observed in cognitive control tasks, following the N2, relating to resource allocation necessary for task performance. Several studies have reported impairments of processes associated with N2 and P3 in ADHD, but the direction in which participants with ADHD differ from HC in these studies was inconsistent. For instance, some studies reported, compared to HC, participants with ADHD showed reduced N2 on successful inhibition trials in a stop-signal task [30] and a Go/No-go task [31], and reduced N2 congruency effect in a flanker task [25]; others did not find N2 differences in stop-signal tasks [32] or reported an increased N2 effect in No-go vs. Go trials in a Go/No-go task [33,34]. Reduced P3 in ADHD has been reported in Go/No-go tasks (see meta-analyses paper [34,35]), a flanker task [36], and an attention network test [37], but not in some other studies (see review paper [38]).

Variability of behavioral and ERP findings might be accounted for by several factors, including participant heterogeneity, sample size, age range, task paradigms and analysis strategies across laboratories [6]. Many studies have failed to report ADHD subtypes and comorbidity; some studies may have been underpowered to detect group differences in behavioral and ERP correlates of performance monitoring. More work is needed to explore whether the inconsistent results can be accounted for by sampling and methodological differences across studies. Moreover, examining how behavior and ERP correlates of performance monitoring correlate with ADHD symptomatology, and whether the relationship may change with age in youth with ADHD, may improve our understanding of performance monitoring processes and their development in ADHD [6]. Few studies have investigated the relationship of behavioral and ERP correlates with symptom severity in children with ADHD. In adults, Marquardt et al. [18] observed that P3 amplitude in high conflict trials was inversely correlated with symptom severity in the ADHD group; Wiersema et al. [20] reported reduced Pe in error trials and P3 amplitude in high conflict trials were associated with more ADHD symptoms; Herrmann et al. [8] compared individuals with low- and high-ADHD symptom scores in a non-clinical population, and observed lower Pe in the group with higher symptoms scores. In adolescents and adults, Michelini et al. [17] found that ADHD symptoms were correlated with congruent errors, reaction time variability, and Pe. Using magnetic resonance imagining, a brain development study of ADHD suggested a brain structural developmental delay in ADHD; this structural development study may be associated with a functional delay [39]. While conflict processing and performance monitoring developed with age in healthy youth, few ERP studies have had a large enough sample size and age range to examine the developmental trajectory of performance monitoring in ADHD [25].

In the present study, we tested a relatively large (number of participants *n* = 77 in each group) sample of children and adolescents with ADHD with a broad age range (8–18 years), and age- and gender-matched HC in an arrow flanker task. Participants’ behavioral performance and ERP correlates of performance monitoring and conflict processing were compared between groups, and these measures were related to ADHD symptom severity measured by the DSM-Oriented ADHD Problems Scale from the Child Behavior Checklist (CBCL) as a continuous measure within and across groups. We hypothesized that youth with ADHD would display slower reaction times, more reaction time variability (RTV), and reduced post-error slowing relative to healthy controls. Considering the inconsistent findings in the ERN, Pe, N2, and P3 in ADHD, we reported ERN and Pe on both error and correct trials and N2 and P3 on both congruent and incongruent trials [6,34]. We focused on difference scores between error and correct trials for the ERN and Pe error effects, along with difference scores between incongruent and congruent trials for N2 and P3 congruency effects, when discussing group differences and developmental alterations in ADHD. We hypothesized reductions in the ERN and Pe error effects and reduced N2 and P3 congruency effects in ADHD, compared to HC. We also hypothesized that behavioral and ERP impairments might be greater with increasing symptom severity within groups. In addition, we investigated whether behavioral and ERP alterations in ADHD vary with diagnostic subtype and age; we hypothesized that there might be a developmental delay in behavioral and ERP indices of performance monitoring in ADHD. 

## 2. Materials and Methods

### 2.1. Participants

There were 77 youth with ADHD (30 female) and 77 age- and gender-matched healthy controls (HC), ranging in age from 8 to 18 years. Patients were recruited through the Section of Child and Adolescent Psychiatry within the Department of Psychiatry at the University of Michigan. Comparison subjects were recruited from the surrounding community. After a complete description of the study, written informed consent was obtained from at least one parent of the participant and written informed assent from the participant. Participants were paid for their interviews and psychophysiological recordings. All tasks and procedures were approved by the University of Michigan Medical School Institutional Review Board. 

All participants were interviewed with the Kiddie Schedule for Affective Disorders and Schizophrenia for School-Aged Children-Present and Lifetime Version (K-SAD-PL) [40] and the Schedule for Obsessive-Compulsive and Other Behavioral Syndromes [41]. Parents completed the Child Behavior Checklist/6–18 (CBCL) [42] and Social Communication Questionnaire (SCQ) [43] about their children. Best-estimate diagnoses were made according to *DSM-5* criteria [44] using all sources of information, including two semi-structured interviews, two parent-report rating scales, four self-report rating scales, and all available clinical records [45]. The clinical records often included outpatient clinic notes, psychological testing results, and teacher rating scales. All participants were evaluated with the Wechsler Abbreviated Scale of Intelligence, Version II (WASI-II), which is normed for individuals ages 6 to 90 [46]. The WASI-II provides a global estimate of overall cognitive abilities with full-scale IQ. The ADHD subtype groups consisted of 23 children with the combined type, six children with the hyperactive-impulsive type, and 48 children with the inattentive type. Eighteen ADHD patients had a comorbid anxiety disorder, eleven patients had comorbid major depression disorder, twelve patients had comorbid oppositional defiant disorder (ODD) and one patient had motor tics that did not interfere with the EEG recording. Participants were excluded if they had a history of intellectual disability, head injury with loss of consciousness, a chronic neurological disorder, or SCQ scores higher than 14. Patients were excluded if they had a lifetime diagnosis of schizophrenia, other psychotic disorder, bipolar disorder, substance-related disorder, conduct disorder, obsessive-compulsive disorder, or anorexia nervosa. HC had no history of a specific axis I disorder. DSM-Oriented ADHD Problems Scale t-scores from the CBCL were used as a continuous measure of ADHD symptoms within and across groups. Symptom counts from the K-SAD-PL (ADHD total score, inattention score, and hyperactive/impulsive score), were used to assess symptom severity in ADHD patients only. Participants with ADHD taking a stimulant (*n* = 43) were asked to stop taking the medication for 48 hours prior to the EEG. Cases and HC were taking no other psychotropic medications. For the error analyses, two participants with ADHD and one healthy control were excluded due to commission of fewer than six errors, leaving a total of 151 participants. 

### 2.2. Procedure

Participants performed a modified Eriksen flanker task in which arrows appeared on a personal computer display with congruent (e.g., →→→→→) and incongruent (e.g., →→←→→) conditions [47]. They were instructed to respond to the central arrow target, while ignoring the adjacent arrows, by pressing one of two buttons indicating the direction of the middle arrow (i.e., right versus left). The stimuli remained on the screen for 250 msec, with an interval of 1500 msec between consecutive trials. 

Each participant was seated 65 centimeters directly in front of the computer monitor and told to place equal emphasis on speed and accuracy in responding. Following 40 practice trials, each subject completed eight blocks of 64 trials for a total of 512 trials. Performance feedback was provided after every block to yield an error rate of approximately 10%, with encouragement to focus on speed if there were fewer than four errors or to focus on accuracy if there were more than 10 errors [48].

### 2.3. Electrophysiological Methods

The electroencephalogram (EEG) was recorded from 64 Ag/AgCl scalp electrodes embedded in a nylon mesh cap, two mastoid electrodes, and two vertical and two horizontal electro-oculographic electrodes using the BioSemi ActiveTwo system (Amsterdam, The Netherlands). Data were digitized at 512 Hz, referenced to a ground formed from a common mode sense active electrode and driven right leg passive electrode (http://www.biosemi.com/ faq/ cms&drl.htm), and re-referenced offline to the average of the two mastoid electrodes. Data were bandpass filtered 0.1–30 Hz using zero-phase shift filters. EEG data were screened using automated algorithms that rejected epochs in which the absolute voltage exceeded 500 μV and epochs containing peak to peak activity greater than 500 μV within 200 msec, with a 100 msec moving window, for midline channels (Fz, FCz, Cz, CPz, and Pz). Ocular movement artifacts were then corrected using a regression-based algorithm [49]. After ocular correction, individual trials were rejected if they contained absolute amplitudes greater than 100 μV, a change greater than 50 μV measured from one data point to the next point, or a maximum voltage difference less than 0.5 μV within a trial in any of the midline electrodes.

### 2.4. Analyses

Behavioral measures included accuracy and mean reaction times (RT) for each participant. Premature responses faster than 150 ms, slow responses longer than 3000 ms, and reaction times beyond three standard deviations from the mean were not considered in the ERP and behavioral analyses. Intra-individual reaction times variability was estimated as the standard deviation across congruent and incongruent trials. Reaction times after errors were evaluated to determine if there were group differences in post-error behavioral adjustments. Accuracy and mean reaction times on correct trials were further analyzed using analysis of variance (ANOVA) with group (ADHD vs. HC) as a between-subject factor and stimulus type (congruent vs. incongruent) as a within-subject factor. 

The ERN was quantified using mean amplitude relative to a pre-response baseline (of −200 to −50 msec). The mean amplitude of the ERN was computed on error trials in a window from 0 to 80 msec following the incorrect response. The correct response negativity (CRN) consisted of the same measure computed on correct trials. The ERN effect was defined as the difference between the ERN and CRN (dERN), calculated by subtracting the CRN from the ERN, since it may isolate activity unique to error processing from activity more broadly related to response monitoring [50]. The Pe was quantified using mean amplitude computed on error trials in a window from 200 to 400 msec following the incorrect response, relative to a pre-response baseline of −200 to −50 msec. The correct Pe consisted of the same measure computed on correct trials. The Pe effect was defined by difference between Pe and Pc (dPe), calculated by subtracting the correct waveform (Pc) from the incorrect (Pe). Both N2 and P3 were quantified using mean amplitude relative to a pre-stimulus baseline of −100 to 0 msec. The mean amplitude of the N2 and P3 were computed on congruent and incongruent correct trials in a window from 300 to 400 ms, and from 400 to 600 msec respectively, following the stimulus onset. N2 and P3 congruency effect were defined by ERP difference between incongruent and congruent trials within corresponding windows. Statistics of ERN, Pe, N2, and P3 were reported at FCz, CPz, FCz, and Pz, respectively, where the maximal mean amplitudes were found, and which were also consistent with previous literature. ERN and Pe amplitude were analyzed respectively with group as a between-subject factor and response type (error vs. correct) as a within-subject factor. N2 and P3 amplitude on correct trials were analyzed with group as a between-subject factor and stimulus type (congruent vs. incongruent) as a within-subject factor. 

One-way ANOVAs were conducted to compare group difference on behavioral and ERP measures. Regression analyses were used to examine (1) correlations of behavioral, ERP measures, and age; (2) group difference on correlations of behavioral, ERP measures and age; (3) behavioral and ERP predictors of ADHD symptom severity. All statistical tests were two-tailed with the alpha level set at 0.05 if not otherwise specified.

## 3. Results

### 3.1. Behavioral Data in Patients with ADHD and Healthy Controls

Participants’ reaction times and accuracy data are presented in Table 1. Participants with ADHD responded more slowly (*p* < 0.01) but as accurately (*p* = 0.77) as HC. Two (congruency: congruent vs. incongruent) by two (group: ADHD vs. HC) repeated-measures ANOVA on correct RT and accuracy revealed main effects of congruency (RT: F (1,152) = 352.4, *p* < 0.001; accuracy: F (1,152) = 470.4, *p* < 0.001) and a trend-level significance of interaction between congruency and group for reaction times (F (1,152) = 3.22, *p* = 0.08), but no such interaction in accuracy (F (1,152) = 0.88, *p* = 0.35). Incongruent trials were completed more slowly and less accurately than congruent trials in both groups. Participants with ADHD tended to have a larger RT congruency effect (86.3 msec vs. 69.5 msec, *p* = 0.08). Moreover, participants with ADHD exhibited smaller post-error slowing (*p* = 0.006) and larger RTV (*p* = 0.005) than HC (Table 1). 

Age in all subjects was negatively associated with overall RT (r = −0.647, *p* < 0.001), RTV (r = −0.620, *p* < 0.001) and RT congruency effect (incongruent minus congruent RT, r = −0.215, *p* = 0.007), and was positively correlated with post-error slowing (*r =* 0.220, *p* = 0.006) and overall accuracy (*r =* 0.264, *p =* 0.001). With age increasing, participants responded faster and more accurately, and showed smaller RT congruency effects and RTV. A correlation of age with post-error slowing was evident in ADHD but not in HC (ADHD: *r =* 0.339, *p =* 0.003; HC: *r =* 0.034, *p =* 0.767; ADHD vs. HC, *p =* 0.012). There was no correlation of age with the accuracy congruency effect. Correlation of age with behavioral measures in individual groups is presented in Table 1. 

### 3.2. ERP Data in Patients with ADHD and Healthy Controls

#### 3.2.1. Response-Locked ERN 

**Figure 1:**
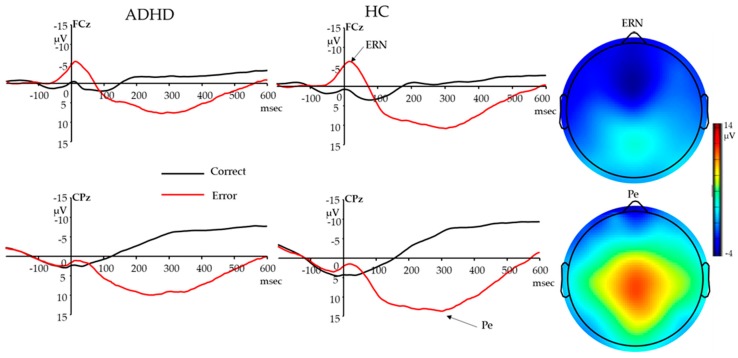
ERN and Pe waveforms for participants with ADHD and HC, and topography for error response (ERN: 0–80 ms; Pe: 200–400 ms; Baseline: −200–−50 ms) in all participants. Responses occurred at 0 msec. ADHD, attention deficits/hyperactive disorder; HC, healthy controls; ERN, error-related negativity; Pe, error positivity.

The two (response type: error vs. correct) by two (group: ADHD vs. HC) repeated-measures ANOVA revealed a main effect of response type (F (1,149) = 118.46, *p <* 0.001) and an interaction between response type and group (F (1,149) = 4.72, *p =* 0.031). Error trials elicited a larger ERN (more negative) than correct trials; the ERN effect (i.e., dERN) was smaller in participants with ADHD than in HC (Table 1; Figure 1). There was no main effect of group (F < 1, *p =* 0.449).

Age was negatively correlated with ERN amplitude (*r =* −0.286, *p <* 0.001) and dERN (*r =* −0.364, *p <* 0.001), and positively correlated with CRN (*r =* 0.167 *p =* 0.040) across all subjects. There was no significant group difference in correlations with age. With age increasing, participants showed a larger ERN, smaller CRN, and larger ERN effect. When covarying age, CRN was negatively correlated with overall RT (*r =* −0.318, *p <* 0.001), RTV (*r =* −0.263, *p =* 0.001), accuracy (*r =* −0.162, *p =* 0.047), and positively correlated with post-error slowing (*r =* −0.204, *p =* 0.012); the ERN effect was positively correlated with overall RT (*r =* 0.311, *p <* 0.001) and RTV (*r =* 0.212, *p =* 0.009) and negatively correlated with post-error slowing (*r =* −0.276, *p =* 0.001). There was no ERN correlation with behavioral performance and no group difference in the correlation between ERN/CRN/dERN and behavioral performance. 

#### 3.2.2. Response-Locked Pe

The two (response type: error vs. correct) by two (group: ADHD vs. HC) repeated-measures ANOVA revealed a main effect of response type (F (1, 149) = 502.62, *p <* 0.001) and interaction between response type and group (F (1, 149) = 8.37, *p =* 0.004). Error trials elicited a larger Pe than did correct trials (Pc); the Pe effect (i.e., dPe) was smaller in participants with ADHD than in HC (Table 1; Figure 1). There was no main effect of group (F = 1.826, *p =* 0.179).

Age was positively correlated with Pc on correct trials (*r =* 0.182 *p =* 0.026); there was no significant correlation of age with the Pe or the Pe effect, and there was no group difference in these correlations with age. When covarying age, the Pe and Pe effect were negatively correlated with overall RT (Pe: *r =* −0.471, *p <* 0.001; dPe: *r =* −0.386, *p <* 0.001), RTV (Pe: *r =* −0.431, *p <* 0.001; dPe: *r =* −0.413, *p <* 0.001), positively correlated with accuracy (Pe: *r =* 0.213, *p =* 0.009; dPe: *r =* 0.313, *p <* 0.001) and post-error slowing (Pe: *r =* 0.249, *p =* 0.002; dPe: *r =* 0.202, *p =* 0.013). There was no Pc correlation with behavioral performance or group difference in correlation between Pe/Pc/Pe effect and behavioral performance. 

#### 3.2.3. Stimulus-Locked N2

**Figure 2:**
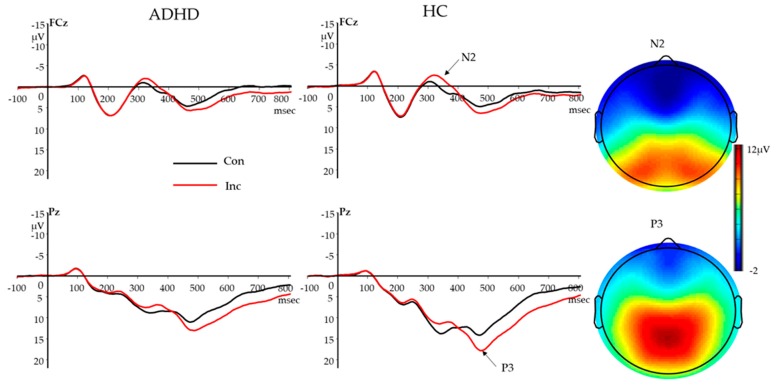
N2 and P3 waveforms for participants with ADHD and HC, and topography for incongruent correct trials (N2: 300–400 ms mean amplitude; P3: 400–600 ms mean amplitude; Baseline: −100–0 ms) in all participants. Stimuli onset occurred at 0 msec. Con, congruent correct trials; Inc, incongruent correct trials; ADHD, attention deficits/hyperactive disorder; HC, healthy controls.

The two (congruency: congruent vs. incongruent) by two (group: ADHD vs. HC) repeated-measures ANOVA revealed a main effect of congruency (F (1,152) = 46.23, *p <* 0.001) and an interaction between congruency and group (F (1,152) = 4.99, *p =* 0.027). Incongruent trials elicited a larger N2 (more negative) than congruent trials; the N2 congruency effect was smaller in participants with ADHD than in HC (Table 1; Figure 2). There was no main effect of group (F < 1, *p =* 0.992).

Age was positively correlated with N2 on both congruent (*r =* 0.395, *p <* 0.001) and incongruent trials (*r =* 0.338, *p <* 0.001), and negatively correlated with the N2 congruency effect (*r =* −0.194, *p =* 0.016). There was no significant group difference in correlations with age. With age increasing, participants showed larger N2 congruency effect (more negative), which was evident in HC but not in ADHD cases (HC, *r =* −0.297, *p =* 0.009; ADHD, *r =* −0.084, *p =* 0.469; ADHD vs. HC, *p =* 0.118). When covarying age, there was no significant correlation or group difference in the correlation between N2 and behavioral performance (all *p*s > 0.09). 

#### 3.2.4. Stimulus-Locked P3

The two (congruency: congruent vs. incongruent) by two (group: ADHD vs. HC) repeated-measures ANOVA revealed main effects of congruency (F (1,152) = 84.24, *p <* 0.001) and group (F (1,152) = 13.12, *p* < 0.001), a significant interaction between congruency and group (F (1,152) = 6.26, *p* = 0.013). Incongruent trials elicited larger P3 than congruent trials. Participants with ADHD had smaller P3 amplitudes, and also showed smaller P3 congruency effects than HC (Table 1; Figure 2). 

Age was not correlated with congruent (*r =* −0.151, *p =* 0.062) or incongruent P3 (*r =* 0.049, *p =* 0.544) amplitudes, but was positively correlated with the P3 congruency effect (*r =* 0.375, *p <* 0.001). There was no significant group difference in the correlation of age with P3 congruency effect. With age increasing, participants showed larger P3 congruency effects. When covarying age, RT on congruent and incongruent trials were negatively correlated with P3 at congruent (*r =* −0.404, *p <* 0.001) and incongruent trials (*r =* −0.432, *p <* 0.001) respectively. The RT congruency effect was negatively correlated with the P3 congruency effect (*r =* −0.171, *p =* 0.034). There was no significant association between accuracy and P3, and there was no group difference in the correlation of P3 with behavioral performance. 

### 3.3. The Association of Behavioral and ERP Measures with ADHD Symptoms and ADHD Subtype

#### 3.3.1. The Association of Behavioral and ERP Measures with K-SAD-PL ADHD Symptoms 

In ADHD, age was negatively correlated with ADHD total score (*r =* −0.385, *p =* 0.001) and hyperactivity/impulsive score (*r =* −0.443, *p <* 0.001). Older youth with ADHD in our sample had reduced symptom severity in total and hyperactivity/impulsive scores; there was no correlation of age with the inattention score (*r =* 0.043, *p =* 0.710). When covarying age, there was no correlation between any ADHD symptom scores (including ADHD total score, inattention score, or hyperactive/impulsive score) and behavioral performance (all *p*s > 0.1). There was no correlation between ADHD symptom scores and ERP measures, except that the P3 congruency effect was found positively correlated with the inattention subscale (*r =* 0.336, *p =* 0.003). 

#### 3.3.2. The Association of Behavioral and ERP Measures with CBCL ADHD Problems Scale Scores

Across all subjects, age was negatively correlated with CBCL ADHD Problems Scale scores (*r =* −0.179, *p =* 0.028). The age correlation with symptom severity was more significant in ADHD patients than in HC (ADHD: *r =* −0.329, *p =* 0.004; HC: *r =* 0.194, *p =* 0.090; ADHD vs. HC: *t* = 2.13, *p =* 0.035). When covarying age, ADHD Problems Scale scores were correlated with overall RT (*r =* 0.330, *p <* 0.001), RTV (*r =* 0.329, *p <* 0.001), post-error slowing (*r =* −0.218, *p =* 0.007) and the RT congruency effect (*r =* 0.161, *p =* 0.049). When age and these significant behavioral measures were included in a backward stepwise regression model, only RTV was found positively correlated with ADHD Problems Scale scores (*b* = 0.031, *p <* 0.001). When covarying age, ADHD Problems Scale scores were correlated with the CRN (*r =* −0.205, *p =* 0.013), ERN effect (*r =* 0.179, *p =* 0.030), Pe (*r =* −0.208, *p =* 0.011), Pe effect (*r =* −0.176, *p =* 0.033), and P3 on congruent (*r =* −0.266, *p =* 0.001) and incongruent (*r =* −0.291, *p =* 0.001) trials. When age and these significant ERP measures were included in a backward stepwise regression model, only P3 on incongruent trials was found negatively correlated with ADHD Problems Scale scores (*b* = −0.339, *p =* 0.004). In the ADHD or HC group alone, there was no correlation of ADHD Problems Scale scores with any behavioral or ERP measures when covarying age. 

#### 3.3.3. Behavioral and ERP Measures Among Different ADHD Subtypes

For patients included in our sample, participants with ADHD inattentive type (14.7 ± 2.8 years) were older than patients with combined type (11.6 ± 2.9, *p <* 0.001), and patients with the hyperactive/impulsive type (12.6 ± 3.9, *p <* 0.001). When controlling age, there were no group differences on any behavioral or ERP measures among the three subtypes of ADHD (all *p*s > 0.1). 

## 4. Discussion

We investigated behavioral and electrophysiological indices of performance monitoring and the association of these indices with age in children and adolescents with ADHD and healthy controls, using an EEG arrow-flanker task. Overall, with age increasing, participants responded faster, more accurately, and less variably, and they showed an increased post-error slowing effect. In addition, they exhibited an increased ERN effect and increased N2 and P3 congruency effects. Children and adolescents with ADHD showed impaired behavioral performance, attenuated error awareness and conflict monitoring. Specifically, participants with ADHD responded more slowly, more variably and had reduced post-error slowing; they showed reduced ERN and Pe effects in error monitoring, and reduced N2 and P3 congruency effects. Impaired behavioral and ERP indices of performance monitoring are consistent with the pattern associated with younger age across groups. Moreover, increased reaction time variability and reduced P3 amplitude in incongruent trials were associated with increased ADHD Problems Scale scores measured by CBCL across all participants. 

The developmental effects on behavioral performance across groups, including faster reaction times, increased accuracy, decreased reaction time conflict, and decreased reaction time variability, are consistent with the literature [51,52]. With increased age and brain maturation, children increase their response speed and improve their attention and ability to resolve conflict. As expected, relative to HC, participants with ADHD responded more slowly, with increased reaction time variability; they also tended to show a larger RT congruency effect. Impairments in these behavioral measures are thought to result from lapses in attention and failures in executive control [17]; the pattern of performance deficits in ADHD is in line with the pattern shown in younger ages across study participants, consistent with the developmental lag model for ADHD [53]. Regarding post-error processes, there was a lack of post-error slowing in ADHD, while HC slowed their response after errors. However, the post-error slowing was positively correlated with age in ADHD, suggesting that the post-error slowing effect in ADHD was not developed until later in adolescence, which further suggests that impairment of performance monitoring in ADHD may be associated with a developmental delay. We did not observe a reduced accuracy among children with ADHD, which may be related to our speed-accuracy instructions to respond quickly enough to maintain a certain number of error trials. Consistently with the previous literature, RTV is found to be larger in ADHD than in HC; among several behavioral indices showing group differences between ADHD and HC, RTV is uniquely associated with continuous measures of ADHD Problems Scale scores, suggesting RTV is a robust marker of ADHD symptoms. 

Error responses elicited a larger ERN and Pe than did correct responses. ERN and Pe effects were larger with faster reaction times, reduced RTV and greater post-error adjustments, suggesting that increased ERN and Pe effects may reflect better performance monitoring and compensatory processing. Consistent with the literature, ERN effects increased with age and Pe effects did not change with age in children and adolescents [54,55]. The reduced ERN effect found in ADHD compared to healthy controls is consistent with most pediatric and adult studies on ADHD [22,56], suggesting an error detection deficit in ADHD. Following early error detection, participants with ADHD showed a reduced Pe effect compared to healthy controls, suggesting alterations in the evaluation of error responses and their motivational significance. Individuals with ADHD may fail to initiate adaptive control processes after errors to make adjustment in the next trial, as demonstrated by diminished post-error slowing. Together with evidence of a larger ERN effect with increasing age, and larger ERN and Pe effects with better performance, the alteration of ERN and Pe effects in ADHD may be associated with a developmental delay in ADHD. 

Incongruent compared with congruent stimuli yielded the typical N2 and P3 amplitude enhancement across groups. Both effects were stronger when participants were older across groups, suggesting larger N2 and P3 effects may reflect enhancements of conflict monitoring with age. While N2 was not associated with behavioral performance, a larger P3 effect was associated with a reduced RT congruency effect. Participants with ADHD showed reduced N2 and P3 effects, indicating problems with conflict monitoring and attention resource allocations. Specially, the attenuated N2 congruency effect in ADHD is consistent with previous findings that N2 is reduced in ADHD and unaffected siblings [25], and in line with the notion that N2 is an index for a general conflict monitoring process. In ADHD, the reduced P3 congruency effect and the reduced P3 amplitude on both congruent and incongruent trials are consistent with previous findings of a general deficit in attention resource allocation [37]. Meanwhile, P3 amplitude on incongruent trials was negatively correlated with CBCL ADHD Problems Scale scores as a continuous measure across groups, further suggesting the cognitive process underlying P3 may serve as a target of intervention to reduce ADHD symptoms. Reduced congruency effects indicated by N2 and P3 in ADHD, together with stronger N2 and P3 effects with age increasing across groups, could also imply a developmental delay in ADHD. 

The current study investigated performance monitoring and its development in ADHD. Our study used a larger sample size (*n* = 77 per group) and broader age range (ages 8–18) than has been typical in the literature, and in addition, our control group was closely matched in age and matched in gender. While the broad age range allowed us to investigate age effects on performance monitoring, the sample was less homogenous and sample size was small for any given age, preventing us from analyzing the effects of other factors, such as gender, comorbidity and medication [6]. Moreover, the current study included participants ranging in age from 8 to 18 years old, though the development of performance monitoring begins earlier and continues later into the life course [49]; further work should evaluate the relationship between brain activation and ADHD across a wider age range. We have interpreted developmental results using a cross-sectional design, which needs to be verified through a longitudinal design. Another limitation of our study is that we included patients with comorbid anxiety, depression and ODD in ADHD group to increase sample size. While comorbid anxiety may have increased ERN in the ADHD group [57], effects of anxiety, depression and ODD comorbidity on different measures of ERPs call for further investigation. 

## 5. Conclusions

This study using ERPs to investigate performance monitoring and its development in ADHD adds to our knowledge of alterations and developmental delays in conflict processing and error monitoring in children with ADHD. Future research should be longitudinal and should include participants across the life span to determine developmental course of performance monitoring and its neural correlates in ADHD. It will also be important to identify behavioral and neural targets for intervention, both for clinical benefit as well as to develop better causal models of the development of ADHD symptoms.

## Figures and Tables

**Table 1:** Demographic, Behavioral and event-related potential (ERP) measures in participants with attention-deficit/hyperactivity disorder (ADHD) compared to healthy controls (HC).

	Mean		Group Difference		Age Correlation (r)		CBCL ADHD Problems Scale Correlation (r), Covarying Age
	ADHD	HC	F	*p*	ADHD	HC	
**Demographic and Clinical Data**							
Age	13.6 ± 3.2	13.6 ± 3.1	0.00	0.982			
IQ	106.3 ± 13.4	110.6 ± 10.2	4.38	0.038			
SCQ	4.0 ± 3.5	1.8 ± 1.8	22.76	0.000			
CBCL_ADHD	65.3 ± 8.3	51.3 ± 3.2	191.71	0.000			
ADHD Symptom Counts from K-SAD-PL							
Hyperactive/Impulsive	3.8 ± 2.8						
Inattentive	7.1 ± 1.6						
Total	10.9 ± 3.1						
**Behavioral Data**							
Overall RT (msec)	580.2 ± 176.0	503.0 ± 142.6	8.92	0.003	−0.668 **	−0.668 **	0.330 **
Overall RTV (msec)	185.6 ± 114.4	135.6 ± 101.7	8.20	0.005	−0.677 **	−0.591 **	0.329 **
Overall Accuracy	90.7% ± 5.9%	90.4% ± 5.6%	0.09	0.769	0.208 ^@^	0.325 **	0.041
Post-error Slowing (msec)	14.5 ± 119.4	57.5 ± 62.9	7.80	0.006	0.338 **	0.034	−0.218 **
Conflict RT (msec)	86.3 ± 62.1	69.5 ± 42.9	3.81	0.075	−0.160	−0.321 *	0.161 *
Conflict Accuracy	10.6% ± 8.0%	10.8% ± 7.6%	0.88	0.350	0.102	−0.056	−0.063
**ERP Data**							
ERN at FCz	−3.20 ± 5.11	−3.78 ± 4.88	0.42	0.474	−0.346 **	−0.223	0.025
CRN at FCz	0.93 ± 4.77	2.41 ± 3.69	4.53	0.035	0.108	0.248 *	−0.205 *
dERN at FCz	−4.13 ± 6.20	−6.19 ± 5.44	4.72	0.031	−0.369 **	−0.368 **	0.179 *
Pe at CPz	8.99 ± 8.82	12.57 ± 9.66	5.62	0.019	0.014	0.047	−0.208 *
Pc at CPz	−5.40 ± 6.50	−6.10 ± 6.19	0.45	0.506	0.268 *	0.095	−0.052
dPe at CPz	14.40 ± 9.37	18.66 ± 8.75	8.37	0.004	−0.173	−0.017	−0.176 *
N2 con at FCz(µV)	0.36 ± 5.57	0.80 ± 5.25	0.23	0.630	0.242 *	0.563 **	−0.003
N2 inc at FCz(µV)	−0.49 ± 5.14	−0.92 ± 4.81	0.30	0.583	0.229 *	0.458 **	0.073
dN2 at FCz(µV)	−0.84 ± 2.17	−1.72 ± 2.55	5.00	0.027	−0.084	−0.297 **	0.148 ^@^
P3 con at Pz(µV)	8.85 ± 5.47	11.58 ± 6.06	8.63	0.004	−0.086	−0.220 ^@^	−0.266 **
P3 inc at Pz(µV)	10.56 ± 6.16	14.57 ± 6.25	16.10	0.000	0.091	0.010	−0.291 **
dP3 at Pz(µV)	1.71 ± 3.20	3.00 ± 3.16	6.27	0.013	0.323 **	0.443 **	−0.113

ERP, event-related potentials; ADHD, attention deficits/hyperactive disorder; HC, healthy controls; SCQ, score from the Social Communication Questionnaire; CBCL_ADHD, the DSM-Oriented ADHD Problems Scale from the Child Behavior Checklist (CBCL); K-SAD-PL, the Kiddie Schedule for Affective Disorders and Schizophrenia for School-Aged Children-Present and Lifetime Version; RT, reaction times; RTV, reaction time variability; Conflict RT, incongruent RT minus congruent RT; Conflict Accuracy, congruent accuracy minus incongruent accuracy; ERN, error-related negativity; CRN, correct-related negativity; dERN, ERN minus CRN; con, congruent; inc, incongruent; dN2, incongruent N2 minus congruent N2; dP3, incongruent P3 minus congruent P3. ** *p <* 0.01, * *p <* 0.05, @ *p <* 0.10.
